# NT2 Derived Neuronal and Astrocytic Network Signalling

**DOI:** 10.1371/journal.pone.0036098

**Published:** 2012-05-02

**Authors:** Eric J. Hill, Cristina Jiménez-González, Marta Tarczyluk, David A. Nagel, Michael D. Coleman, H. Rheinallt Parri

**Affiliations:** 1 Aston Research Centre into Healthy Ageing (ARCHA), Aston University, Birmingham, West Midlands, United Kingdom; 2 School of Life and Health Sciences, Aston University, Birmingham, West Midlands, United Kingdom; Tokyo Medical and Dental University, Japan

## Abstract

A major focus of stem cell research is the generation of neurons that may then be implanted to treat neurodegenerative diseases. However, a picture is emerging where astrocytes are partners to neurons in sustaining and modulating brain function. We therefore investigated the functional properties of NT2 derived astrocytes and neurons using electrophysiological and calcium imaging approaches. NT2 neurons (NT2Ns) expressed sodium dependent action potentials, as well as responses to depolarisation and the neurotransmitter glutamate. NT2Ns exhibited spontaneous and coordinated calcium elevations in clusters and in extended processes, indicating local and long distance signalling. Tetrodotoxin sensitive network activity could also be evoked by electrical stimulation. Similarly, NT2 astrocytes (NT2As) exhibited morphology and functional properties consistent with this glial cell type. NT2As responded to neuronal activity and to exogenously applied neurotransmitters with calcium elevations, and in contrast to neurons, also exhibited spontaneous rhythmic calcium oscillations. NT2As also generated propagating calcium waves that were gap junction and purinergic signalling dependent. Our results show that NT2 derived astrocytes exhibit appropriate functionality and that NT2N networks interact with NT2A networks in co-culture. These findings underline the utility of such cultures to investigate human brain cell type signalling under controlled conditions. Furthermore, since stem cell derived neuron function and survival is of great importance therapeutically, our findings suggest that the presence of complementary astrocytes may be valuable in supporting stem cell derived neuronal networks. Indeed, this also supports the intriguing possibility of selective therapeutic replacement of astrocytes in diseases where these cells are either lost or lose functionality.

## Introduction

There is currently great scientific and medical interest in the potential of tissue grown from stem cells. To control stem cell differentiation to produce specific tissues would enable the grafting and possible replacement of diseased and damaged organs. In addition, it would also present opportunities for generating model systems for drug screening and toxicological testing which would be expected to be more relevant to human outcomes than animal based tissue preparations.

The NTERA-2 (NT2) cell line is derived from a human male germ cell carcinoma. It is one of the best documented stem cell lines, as it can be differentiated into neuronal cells (NT2N) by retinoic acid treatment [1], [2], [3], [4]. NT2.D1 cells are similar to the murine P19 embryocarcinoma cell line [5]. P19 cells can also be differentiated using RA to produce neuronal cells which appear within the first few days of treatment and astrocytes by day 10. Both cells have the advantage that the cells are immortal thus allowing for the creation of almost unlimited amounts of material for differentiation. These cells are easy to grow and maintain in the undifferentiated state but they can also be efficiently induced to differentiate by simple manipulation of the culture conditions. However, as the NT2.D1 cells are human in origin they have a distinct advantage over the murine P19 cells for studying aspects of human neuronal systems and also regarding therapeutic potential, to the point where they have been used in human neural transplantation trials in patients following stroke [6].

Characterisation of NT2N cells through immunocytochemical staining has revealed their neuronal features, including expression of MAP2, the dendritic filament marker. Within the NT2N population, heterogenous sub-populations are also produced; indeed, putative dopaminergic cells have been identified by immunocytochemical or RT-PCR identification of tyrosine hydroxylase, cholinergic neurons by cholineacetyltransferase, GABAergic neurons by GAD staining and glutamatergic neurons by staining for vesicular glutamate transporters [7]. Great focus has also been placed on defining the electrophysiological properties of these cells, since functional neurons must also have the ability to generate action potentials and sustain neurotransmitter release, as well as respond to neurotransmitters. These are therefore prerequisites for NT2N to be considered functional neurons. Crucially, NT2N cells have been found to generate action potentials on depolarisation [8], [9], and they also express the high voltage activated calcium channel currents pharmacologically classified as L, N, P/Q and R [10] and calcium activated BK channels, which are involved in neuronal hyperpolarisation following action potential firing [11]. Receptors which would detect different released neurotransmitters are also present on NT2Ns including NMDA receptors [12], nicotinic acetylcholine receptors [13] and GABA_A_ receptors [14].

The potential competence of stem cell derived neurons to integrate *in vivo* depends on the ability to form synaptic connections with each other and with native neuronal networks. Importantly, functional synapses have been identified in NT2N cultures [8] and spontaneous excitatory and inhibitory currents likely to correspond with glutamatergic excitatory postsynaptic currents and GABAergic inhibitory postsynaptic currents have also been recorded [15]. Furthermore, when grown on microelectrode arrays, NT2N cells also form functional networks which exhibit spontaneous activity, although the activity was sparser and less synchronised than primary cultures of rat cortical neurons [6], [16].

A notable revolution that has occurred within neuroscience in the past 10 years involves the realisation of the role of glial cells in brain function, where astrocytes are active partners in synaptic transmission. Previously, astrocytes that ensheath synapses in the brain were considered to primarily conduct housekeeping functions such as glutamate uptake and potassium balance [17]. The current hypothesis, encapsulated by the concept of the tripartite synapse [18] posits that astrocytes sense neuronal synaptic transmitter release and in turn release gliotransmitters which can feedback to modulate neuronal activity.

These newly realised astrocytic roles in the brain have fundamental implications within the context of stem cell derived neuronal networks. If the aim of stem cell neuroscience is to generate functional neuronal networks that behave as networks do in the brain, then it becomes clear that we must include and understand all the cellular components that comprise that network, and which are important to support synaptic integrity and cell to cell signalling. A prominent overlooked cell in this regard is the astrocyte.

Following injection into mouse brains, NT2 cells differentiate into neuronal and glial cell types [19]. However, despite this very little is known about the functional properties or potential of NT2A cells. It is however, known that NT2N cells generally survive for 2–3 months in normal media [4], whereas mixed NT2.N/A cultures typically survive for 6–9 months, clearly demonstrating the importance of neuronal-astrocytic interactions for neuronal maintenance [4], [20], [21]. Growing NT2N cells on astrocytes is also known to be favourable to synapse formation [8]. It has also been found that NT2A cells are coupled via connexin 43 containing gap junctions [20]. In addition, in our laboratory we have used the NT2N/NT2A co-culture system, to demonstrate that astrocytes confer significantly increased neuronal resilience to chemical toxicity and that neurons and astrocytes display markedly differential sensitivities to a number of CNS toxins [22], [23]. Such findings therefore provide evidence of the distinct functionality of NT2A cells.

In this study, we therefore utilised culture methods developed to produce pure, and mixed cultures of NT2N and NT2A cells to investigate the functional properties of NT2A cells, the function of NT2N networks in the presence of NT2A cells and interactions of the astrocyte network with the neuronal network. We found that NT2 cells differentiate into distinct cell types in culture with functional characteristics consistent with their neuronal and astrocytic cellular classification. We also found that neuronal networks signal to astrocytes, and that astrocytic networks communicate via gap junction mediated and gliotransmitter signalling. These findings illustrate that stem cell derived NT2A cells possess all of the tested functional competencies of *in vivo* astrocytes.

## Results

**Figure 1:**
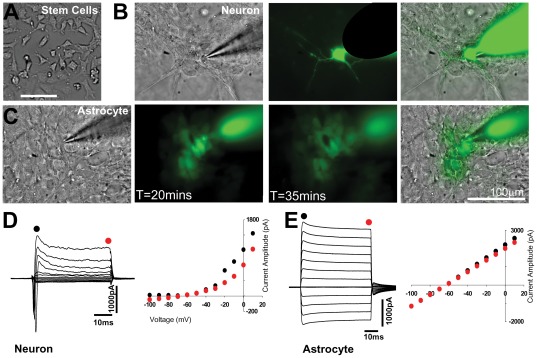
Distinct functional properties of NT2 neurons and astrocytes. **A**. DIC image of undifferentiated NT2 stem cells displaying undifferentiated morphology. **B**. Leftmost panel shows DIC image of a patched neuron in a cluster. Central image shows the same cell excited at 488 nm to reveal neuronal morphology from the patch pipette filling by Alexa 488. Rightmost panel superimposes the green and DIC channels, illustrating the pathway of the neuron's processes within and efferent from the cluster. **C**. Leftmost panel shows DIC image of a patched astrocyte, image to right shows a fluorescent image of the field taken 20 minutes after patch clamping an astrocyte with Alexa 488 containing pipette. Many astrocytes can be seen to be filled, and displaying a “tile-like" morphology. Middle panel shows an image taken 15 minutes later illustrating further spread of the Alexa 488 dye. Righmost panel superimposes the green and DIC channels. **D**. Current responses elicited by voltage steps in a patch clamped neuron. Depolarisations elicit a transient inward current and sustained inactivating outward current. The maximal and steady state amplitude of the outward component (Black and Red symbols respectively) are plotted to the right illustrating an outwardly rectifying nature. **E**. Current responses in an astrocyte elicited by voltage steps, displaying lack of transient components. The plot of instantaneous and steady state current reveal a linear I–V relation. Cells were used within 14 days of differentiation.

To investigate the functional characteristics of NT2 derived astrocytes and neurons. NT2 stem cells were differentiated as previously described [24]. In their undifferentiated state stem cells displayed their characteristic amorphous structure devoid of processes (Fig. 1A). Following treatment with retinoic acid, two cell populations were generated in culture. NT2 Astrocytes (NT2A) preferentially occupied the base of the culture dishes forming planar associations, whilst NT2 neurons (NT2N) formed clusters above (Fig. 1B). Filling of single cells via a patch pipette with Alexa 488 further revealed distinct morphological characteristics, with neurons having small diameter somas (8.2±0.41 µm, n = 15) and long processes (>100 µm), whilst astrocytes had somas of diameter 18.8±1.2 µm (n = 12) and no visible elongated processes. (Fig. 1B, C). Patch clamp recordings revealed that NT2 neurons and astrocytes had distinct electrophysiological properties and expressed different voltage dependent currents. NT2Ns had an input resistance of 65±11.2 MΩ (n = 5). In voltage clamp recording mode they exhibited a fast inward transient current on depolarisation followed by an outward current that had inactivating and sustained components (Fig. 1D). The current –voltage relationship of the NT2 neurons revealed an outwardly rectifying current with little inward current to hyperpolarisation. In contrast, NT2As had an input resistance of 30.7±6.6 (n = 5, P<0.05) MΩ. NT2As displayed no transient currents, and large currents were elicited in depolarising and hyperpolarising directions giving a linear current-voltage relationship (Fig. 1E).

**Figure 2:**
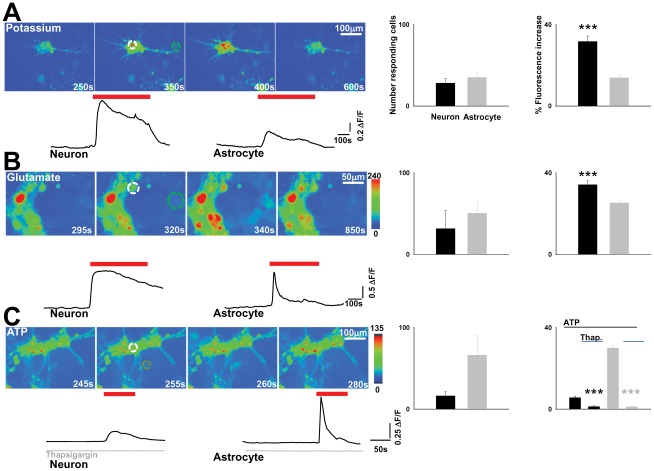
Neurons and astrocytes respond to chemical stimulation. **A**. Images taken at different stages during an experiment illustrating responses to increased potassium. Traces below plot fluorescence over the time course for indicated neuron (white circle) and astrocyte (green circle). Bargraphs to the right display the number of neurons and astrocytes responding with calcium elevations to potassium and the amplitude of fluorescence increase in the two different cell populations. **B**. Images illustrating calcium elevations in response to glutamate. Responses from the neuron (white circle) and astrocyte (green circle) are plotted in the traces below. Bargraphs to the right display the number of neurons and astrocytes responding with calcium elevations to glutamate and the amplitude of fluorescence increase in the two different cell populations. **C**. Images illustrating calcium elevations in response to ATP. Responses from the neuron (white circle) and astrocyte (green circle) are plotted in the traces below. Grey traces show responses following thapsigargin treatment. Bargraphs to the right display the number of neurons and astrocytes responding with calcium elevations to ATP and the amplitude of fluorescence increase in the two different cell types in control conditions and following thapsigargin treatment. Cells were used within 14 days of differentiation.

In neurons, electrical activity is transduced to a biochemical signal by the elevation of calcium entry via voltage gated calcium channels. Neurotransmitter receptor activation is also coupled to calcium increases. Although astrocytes are electrically non-excitable, their excitability is defined by variations in intracellular calcium [17]. We therefore used a calcium imaging approach to probe functional signalling in the NT2 derived cells. Cultures were loaded with the cell permeable calcium indicator fluo-4, and activity imaged over time (Fig. 2). Increasing extracellular potassium concentration to depolarise neurons and astrocytes induced calcium elevations in both neurons (28.2±5.34, n = 4 fields) and astrocytes (Fig. 2A)(35.01±6.05, n = 4 fields) with a greater calcium increase in neurons (31.68±2.67%) compared to astrocytes (13.84±1.04, P<0.0005), consistent with depolarisation induced activation of voltage gated calcium channels.

The main neurotransmitter in the brain mediating chemical signalling between neurons and from neurons to astrocytes is the amino acid glutamate, and the main transmitter molecule implicated in astrocytic signalling is ATP [25]. Indeed, application of 100 µM glutamate resulted in calcium elevations in neurons (31.66±22.12, n = 6) and astrocytes (50.55±14.41) with similar magnitude fluorescence increases (neurons: 34.22±1.98% astrocytes: 25.26±1.11 Fig. 2B). Application of ATP also elicited calcium elevations in both neurons and astrocytes (Fig. 2C) however with a greater magnitude in astrocytes (Fig. 2C, neurons 5.63±0.69%, astrocytes 30.23±1.29%, n = 4). ATP induced calcium elevations were inhibited by the intracellular store depletor thapsigargin (neurons: 1.27±0.35%, astrocytes: 1.14±0.84%, P<0.0005) consistent with these being the main calcium stores for signalling in astrocytes.

**Figure 3:**
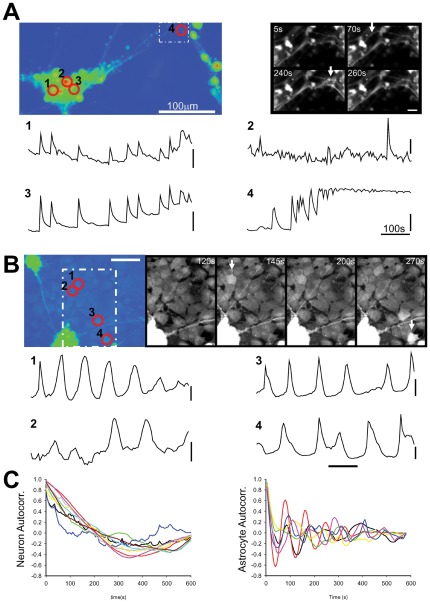
Stem cell derived neurons and astrocytes display spontaneous activity. **A**. Image on the left displays an NT2N neuronal aggregate with extending processes. The white dotted area is displayed expanded to the right at indicated time points during the experiment. White arrows indicate processes that become active during acquisition. Red numbered circles indicate exemplar active cells with corresponding fluorescence timecourse displayed below. Neurons can display synchronised activity (1,3). Propagating activity from distant processes can also be detected (4). **B**. Image on the left displays an NT2N-A coculture. Spontaneously active astrocytes are circled and numbered. White dotted area is displayed expanded to the right at indicated time points during the experiment. White arrows indicate active astrocytes. Fluorescence time courses from the astrocytes are illustrated below, indicating regular calcium oscillations. **C**. Autocorrelation analysis of neurons from experiment A and astrocytes from experiment B. Scale bars A-1-4: 2, 0.5, 10, 10%. B-1-4: 5, 5,10, 10%. Cells were used within 14 days of differentiation.

Neurons and astrocytes studied in *in vivo* and in *ex vivo* brain slice preparations exhibit spontaneous activity [26], [27], [28]. Conducting time lapse imaging over a 10 minute period without stimulation revealed spontaneous calcium activity in neurons (16.01±6.28) and astrocytes (26.81±13.51, n = 5)(Fig. 3.) showing that in the absence of external input the NT2 cultures are capable of generating activity. The features of the observed activity were different between the cell types. Neurons within clusters displayed sporadic activity which could be synchronised with other neurons in the cluster (Fig. 3A). Activity could also be seen to propagate along neuronal processes to distant clusters. Astrocytes however, often exhibited regular “pacemaker" type oscillatory activity (Fig. 3B). The mean inter-event interval of neuronal and astrocytic activity was 63.15±9.37 s (n = 54 neurons, 3 preparations) and 93.1±6.3 s (n = 108 astrocytes, 4 preparations). The rhythmicity of astrocytic oscillations compared to neuronal activity was revealed by autocorrelation analysis (Fig. 3C).

**Figure 4:**
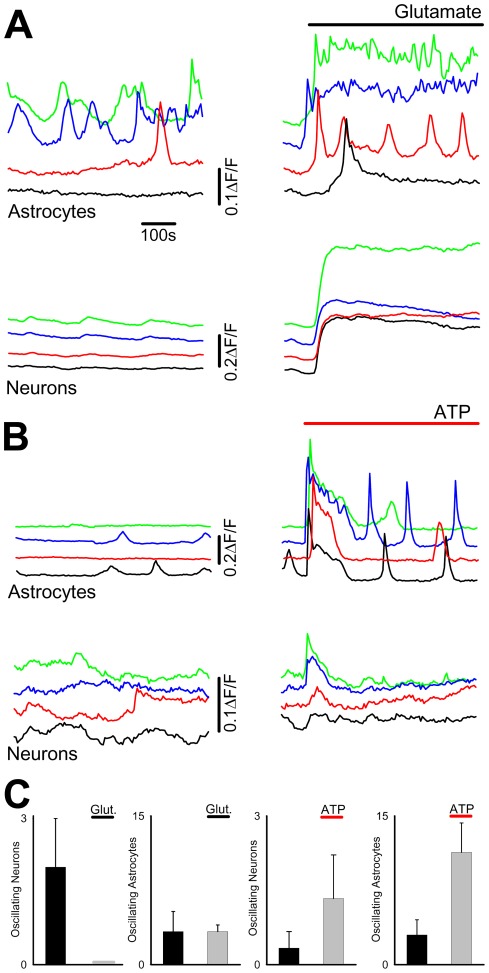
Sustained transmitter exposure in eliciting calcium oscillations. **A**. Fluorescence time courses from astrocytes and neurons in the same experiment before (left panels), and following (right panels) sustained application of glutamate. Activity in the same cell is indicated by colour. **B**. Activity in astrocytes and neurones before (left panels) and following (right panels) application of ATP. **C**. Bargraphs summarising results from a number of experiments illustrating the effect of sustained glutamate and ATP application on the number of astrocytes and neurons expressing calcium oscillations. Cells were used within 14 days of differentiation.

Acute application of glutamate and ATP (Fig. 2) demonstrated that NT2N and NT2A cell types expressed functional responses to the two transmitters. To determine whether these transmitters could participitate potentially in longer term signalling, such as spontaneous oscillations seen in Fig. 3, the effects of transmitters were determined over longer periods (Fig. 4). Application of glutamate elicited transient calcium elevations as previously observed, which were sustained in neurons (Fig. 4A) but transient in astrocytes. Glutamate did not affect the number of astrocytes displaying repetitive calcium oscillatory activity (control: 3.3±2.20, glutamate 3.33±0.67, n = 3,)(Fig. 4A,C), but reduced neuronal oscillatory activity (control:2±1, glutamate: 0±0, n = 3) seemingly abrogating network activity.

Similarly, sustained ATP stimulation of neurons did not induce oscillatory activity (control: 0.33±0.33, ATP: 1.33±0.88, n = 3), whilst ATP led to the generation of regular calcium oscillations in astrocytes (control: 3±1.52, ATP: 11.3±2.9, n = 3, P<0.05)

**Figure 5:**
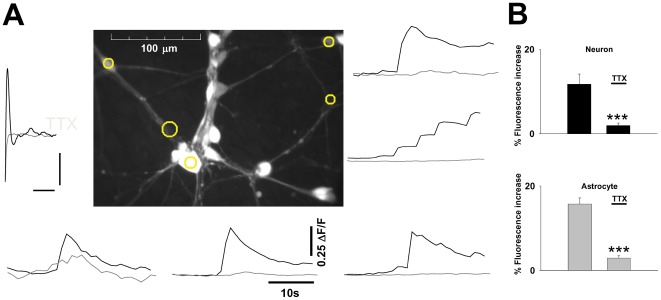
NT2 neurons support network activity. **A**. Centre image shows neuronal cell bodies and processes. Plots of fluorescence over time from the circled areas are displayed to the right and below. Black traces illustrate response to electrical stimulation in control conditions and grey traces following TTX application. **B**. Bargraphs summarise calcium elevation responses of neurons and astrocytes for a number of experiments. Scale bar in A, 5 mV, 2 ms. Cells were used within 14 days of differentiation.

The NT2 neurons in our cultures exhibited voltage gated sodium currents (Fig. 1), and individual cells exhibited spontaneous calcium elevations. The step depolarisation of NT2 neurons in current clamp mode elicited transient spikes (13.7±1.2 mV, n = 4) which were blocked by TTX, identifying them as Na^+^ channel dependent action potentials. A greater amplitude than this might be expected to be required to sustain synaptic transmission, therefore to determine whether the NT2 neurons were capable of signalling synaptically during electrical activity in the NT2 networks, we stimulated with a bipolar electrode. Stimulation resulted in calcium elevations in neuronal cell bodies and processes (Fig. 5) throughout the observed network (11.71±2.41%, n = 5). These were blocked in the presence of TTX (1.94±0.62%, P<0.0005) indicating that signal propagation throughout the neuronal network is indeed action potential dependent. Astrocytic calcium elevations in the vicinity of the neurons were also evoked by electrical stimulation (15.69±1.46%, n = 5), and these were blocked by TTX (to 2.89±0.57%, P<0.0005). Since astrocytes do not express TTX sensitive currents this is consistent with electrically induced neuronal neurotransmitter release activating astrocytic receptors as seen in brain slice preparations [29], [30] and in vivo [31].

**Figure 6:**
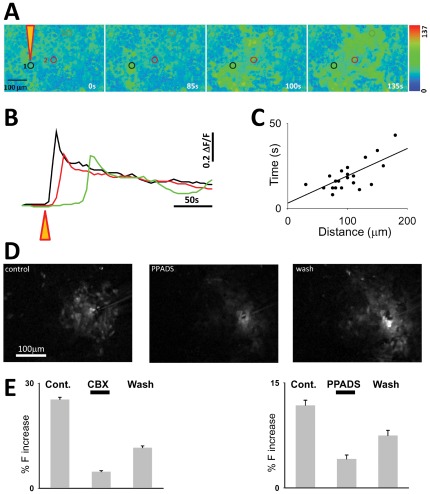
Calcium wave propagation in astrocytic networks. **A**. Images showing a field in a pure NT2 astrocyte culture loaded with Fluo-4. Following image at t = 0 s, a mechanical stimulation was applied at point indicated by orange triangle tip. Subsequent images display the progression of a calcium wave in surrounding astrocytes. **B**. The time course of fluorescence in the circled astrocytes at different distances from the origin. **C**. Plot of wave distance with time for a number of experiments fitted with a linear regression. **D**. Maximum projection images showing extent of wave propagation in control, with PPADS and following PPADS wash off. **E**. Bargraphs summarising a number of experiments where fluorescence was measured in astrocytes during wave propagation, and the effect of CBX and PPADS. Cells were used within 14 days of differentiation.

Whilst the main mechanism of information transfer between neurons is the synaptic release of chemical neurotransmitters, astrocytes in the brain are coupled via gap junctions [32]. These enable the inter-astrocytic transfer of metabolically important molecules such as glucose [33], and possible signalling molecules such as IP3 [34]. Another feature of astrocytic syncytia is that they can propagate calcium waves in response to chemical and mechanical stimulation [35]. We tested this property in pure cultures of NT2 astrocytes. A mechanical stimulation was applied using a micromanipulator controlled patch pipette placed above a single astrocyte, and lowered to stimulate a single astrocyte. Following stimulus, a calcium elevation was induced in the stimulated astrocyte which then propagated through the astrocytic field, by an apparent sequential recruitment of adjacent astrocytes (Fig. 6A,B). A linear regression of distance against time of peak calcium elevation yielded a slope of 3.1 and r^2^ = 0.5. The mean velocity calculated from the distance from stimulus origin and peak of calcium elevation was 5.59±0.43 µm/s (n = 25, 4 preparations)(Fig. 6C), similar to that seen from studies of astrocytic primary culture and signal propagation in brain slice [26]. The propagation was at least partly dependent on gap junction communication since the amplitude of calcium elevations at astrocytes >50 µm distant (25.33±0.66%, n = 3) from the origin astrocyte was reduced in the presence of the gap junction blocker carbenoxolone (CBX) (4.72±0.33% P<0.0005). This recovered on CBX wash-off (11.60±0.51% P<0.0005). In addition to gap junction contribution to calcium wave propagation [36], [37], the gliotransmitter ATP is implicated in mediating waves [35]. We therefore investigated the effect of purinergic P2X and P2Y receptor antagonist PPADS (100 µM) on propagation. The presence of PPADS resulted in a reduction in the amplitude of measured astrocytic calcium elevations in the pathway of the wave from 11.79±0.72% to 4.14±0.54% (P<0.0001) which recovered on wash (7.49±0.74, n = 76 astrocytes, 4 preparations), consistent with a restricted extent of wave propagation (Fig. 6D,E).

## Discussion

It is now becoming clear from *in vitro* brain slice and *in vivo* experiments, that for synaptic communication in the brain astrocytes and neurons form a functional unit, known as the ‘tripartite synapse’ [18]. This challenges the traditional neurocentric view of brain function and contends that it is the interactions between astrocytes and neurons that govern how the brain operates.

In the same way, to understand stem cell-derived neurons, and their potential cellular and network functions, as well as understanding how to optimise this function, most certainly requires their study within the context of their association with astrocytes. It may be reasonably expected that the full functional potential of stem cell derived neuronal networks will most likely be realised when they differentiate and develop in the presence of their astrocytic counterparts. If these models are to be applied to the investigation of human neuronal function, it is thus essential that the neuron-astrocyte relationship is sufficiently representative of anatomical and functional reality to permit rational hypothesis testing. In this study we therefore used selective methods to generate either cultures containing one of the cell types or cultures containing both, in contrast to the standard methods used to generate pure neuronal cultures utilised in neuro-centric focused studies. Neurons and astrocytes in the brain have distinct electrophysiological and morphological properties. Neurons extend axons to synapse on other neurons and possess dendritic processes onto which other neurons synapse. Neurons notably express voltage gated sodium channels that underlie action potential firing which depolarise the presynaptic terminal to allow calcium entry, neurotransmitter release and so initiate synaptic information transfer. Astrocytes, on the other hand, display electrophysiologically passive properties and have fine, restricted processes. In NT2 co-cultures there was a clear morphological distinction between NT2N and NT2A cells. NT2N cells formed clusters which were often connected by long tracts. NT2A cells formed a layer below the clusters and displayed a tile like morphology characteristic of cultured astrocytes. Filling individual cells with fluorescent Alexa 488 revealed the stereotypical neuronal morphology of NT2N cells in clusters, with apparent dendritic processes, and longer axonal process which could extend into the inter-cluster tracts. This indicates that morphologically, NT2 neurons have the structural capability to signal between and possibly within clusters. Calcium imaging experiments confirmed the signalling role of these processes.

Filling of astrocytes, revealed stereotypical cellular morphology, as well as a time dependent-filling of adjacent astrocytes, a pattern not seen with neuronal filling. This confirms previous reports that NT2A cells are physically coupled via gap junctions [20], as astrocytes are in the brain. Such coupling has most recently been suggested to be involved in controlling the supply of glucose to neurons in the hippocampus [33]. It is therefore possible that they could be fulfilling a similar function for NT2N cells. It has also been shown that embryonic stem cell derived astrocytes can integrate into native rat syncytia [38]. Our patch clamp recordings confirmed that neurons displayed voltage activated currents, notable fast sodium transients consistent with recordings from a previous study [9] whilst astrocytes exhibited a passive profile.

The main neurotransmitter in the brain is glutamate, whilst ATP is also a transmitter at some synapses [39]. ATP and glutamate are also gliotransmitters, released from astrocytes in a calcium dependent manner [35], [40]. Astrocytic glutamate release is known to modulate synaptic transmission in the hippocampus [41], whilst ATP release and its possible degradation to adenosine are also implicated in synaptic modulation [42]. ATP release from astrocytes has also been shown to act in a paracrine manner in propagating astrocytic excitability which can affect heterosynaptic depression [43]. Our findings thus show that in addition to reported neuronal receptor expression [12], [13], [14], [44], NT2 astrocytes also express transmitter receptors providing them with the potential to participate in astrocyte-neuron and astrocyte-astrocyte communication *in vitro* and in brain grafts.

The main way that astrocytes sense neuronal activity in the brain is by responding to synaptically released neurotransmitters. *In vivo* experiments have shown that sensory stimulation in the form of rodent whisker stimulation [31] or ferret visual stimuli can elicit astrocytic calcium elevations. Indeed, astrocytes in the visual cortex exhibit orientation selectivity [45]. Our results show that neuronal stimulation also leads to astrocytic calcium elevation and that blocking neuronal transmission with TTX blocks astrocytic responses, indicating that NT2 astrocytes can sense NT2 network synaptic activity.

A widely reported feature of astrocytes in the brain is the expression of spontaneous calcium activity [26], [28], this likely reflects many processes, including reaction to ongoing synaptic activity. However, astrocytes also display calcium oscillations when neuronal activity is blocked, and these oscillations can display regular pacemaker patterns [46]. We also found this activity in NT2 astrocytes, which differed from the calcium oscillations seen in neurons. The role of such activity is unknown, although it is possible that these oscillations drive the release of signalling molecules such as gliotransmitters, cytokines or trophic factors. Our findings that sustained exposure to ATP induced astrocytic calcium oscillations indicates that ATP may be involved in the generation of such activity in NT2A cells and also that transmitters can induce longer term activity patterns in these cells which depending on the subsequent astrocytic output could have significant functional effects on NT2N neuronal networks.

A standard approach of monitoring neuronal population activity is by using microelectrode arrays, and this approach has also been used to probe NT2N neuronal network activity. One disadvantage of this approach is, however, that activity in only a small proportion of the actual network is recorded due to limitations of electrode spacing. To address this in the living brain, researchers have developed complex multiphoton imaging and sampling methods [47]. In this study, we also show that for monitoring large populations of NT2 neurons and astrocytes in culture, calcium fluorescence imaging is an effective alternative or complement to electrophysiological recording.

The results from this study show that in coculture, NT2N and NT2A cells are distinct cell types which express the expected functional properties of neurons and astrocytes. Whilst it has been established that NT2 neurons can form functional networks, the findings of this study extends the knowledge of stem cell derived human neuron-astrocyte cultures to show that NT2 astrocytes exhibit functional calcium elevations to exogenously applied neurotransmitters, as well as to synaptic stimulation. NT2-Astrocytes also respond to mechanical stimulation and can propagate activity through the astrocyte syncytium, as observed *in vivo*. Overall, the findings indicate that NT2 neuron and NT2 astrocyte networks can communicate, and so have the potential to interact in a tripartite manner as is seen *in vivo*. This study therefore lays the foundation for further development of stem cell derived neurons and astrocytes into therapautic cell replacement and human toxicology/disease models.

## Materials and Methods

### Generation of NT2N and NT2A cells and Cell Culture

Human NT2/D1 cells were kindly donated by Prof. P.W Andrews (University of Sheffield, UK). To generate pure NT2N NT2N and NT2NA cultures, NT2 cells were differentiated with 10 µM Retinoic Acid for 4 weeks. Subsequent to plating, cells were treated with media containing mitotic inhibitors [1 µM cytosine arabinoside (ARAC) for 1 week, 10 µM fluorodeoxyuridine (FDU) and 10 µM uridine (U) for 3 weeks to produce a neuronal monoculture. To derive a neural-astrocytic co-culture NT2 cells were also differentiated with 10 µM Retinoic Acid for 4 weeks, but with anti-proliferative treatment with media containing a lower concentration of mitotic inhibitors according to the method described in [24]. Following differentiation with 1×10^−5^ M retinoic acid for 4 weeks, NT2 cells were seeded into CellBIND 12-well plates (Corning, USA) at a density of 2.25×10^6^ cells/well. To suppress the proliferation of non-neural cell types, mitotic inhibitor treatment was performed. Cells were treated with media containing 0.1 µM cytosine arabinoside for 1 week, 3 µM fluorodeoxyuridine and 5 µM uridine for 4 weeks to generate mixed cultures of neurons and astrocytes. Upon replating of RA treated cells, neuron-like cells with small neurite outgrowths could be observed growing out from larger clusters of cells. Sandhu et al 2002 [4]demonstrated that cells replated after RA treatment could be mechanically treated to remove these loosely attached neuronal cells to establish astroglial cultures that matured over a period of 6 passages to produce 99% pure astrocytic cultures without the use of MI. These cells have morphological characteristics that are consistent with fibrous or stellate astrocytes and protoplasmic or polygonal astrocytes, respectively.

In our method, upon replating these clusters by mechanical dissociation and subsequent MI treatment we were able to suppress the growth of immature astrocytes/astrocytic precursors using 1 µM ARAC and 10 µM FDU and U to produce pure neuronal cultures. However, if cells were replated into media containing lower concentrations of mitotic inhibitors then these immature astrocytes were able to proliferate and differentiate into GFAP positive cells after 28 days that no longer divided. This would suggest that after 28 days in RA, immature astrocytes/astrocytic precursors are present in the cultures and that their growth is suppressed by treatment with high concentrations of MI. After 28 days in lower concentrations of MI a co-culture of neuronal and astrocytes was established. However, in the co-culture these cells were not inhibited but differentiated into GFAP positive cells with morphological features of mature astrocytes. The putative neuronal cells present after 28 days RA treatment, continued to extend neurite-like processes during MI treatment, irrespective of the differentiation strategy used. After 28 days these cells displayed distinct mature neuronal morphology.

The proportion of cell types produced by this method in this study were in agreement with previously published values (33±4% neurons and 63±4% astrocytes) [23]. NT2A cultures in our hands typically survive >9 months (unpublished data).

Once differentiated cells were shown to no longer divide they were therefore maintained in Dulbecco's Modified Eagle Medium (D-MEM)-high glucose supplemented with 10% heat inactivated foetal bovine serum (FBS, Invitrogen), 100 units/ml Penicillin, 100 µg/ml Streptomycin (Invitrogen). All experiments were performed on cells within 2 weeks of the differentiation process.

### Electrophysiology

The recording chamber and manipulators were mounted on a moveable top plate platform (MP MTP-01, Scientifica, UK). Patch clamp recordings were made using pipettes (2–4 MΩ) containing an internal solution of composition (in mM): KMeSO_4_ 120, HEPES 10, EGTA 0.1, Na_2_ATP 4, GTP 0.5. and Alexa-hydrazide 488 0.1, for morphological identification. Currents were recorded using a Multiclamp700B amplifier, digitized with a Digidata 1440A and acquired and analysed using PClamp (Molecular Devices, Ca. USA). Voltage clamp recordings were made at indicated potentials. Cells with ≥20% change in access resistance were excluded. Acquired data was analysed using the Clampfit routine of PClamp. Data was exported to Sigmaplot (Jandel) for further analysis and plotting.

### Synaptic stimulation

Synaptic stimulation was achieved with a computer controlled constant current isolated stimulator (STG1002, Multichannel Systems, Germany) and bipolar electrodes, which were placed typically >200 µm from the imaged field.

### Fluorescence imaging

In these experiments, cultures were loaded with Fluo-4 or Fura-2 AM (Molecular Probes, Eugene, Or., USA). This was done by incubating for ∼50 min at 37°C with 5 µM of the indicator dye and 0.01% pluronic acid. The recording chamber and manipulators were mounted on a motorized moveable bridge (Luigs and Neumann, Germany) and fluorescence dyes were excited using an Optoscan monochromator system (Cairn, UK), fitted to a Nikon FN1 upright microscope; filter cubes were obtained from Chroma (Chroma VT, USA). Images of areas of 444 µm×341 µm were routinely acquired every 5 s with a ×20 objective lens (NA = 0.8) or 218 µm×168 µm with a ×40 lens (NA = 0.9) using an ORCA ER CCD camera (Hamamatsu) and analysed using Simple PCI software (Compix Hamamatsu, Digital Pixel, UK). Autocorrelation analysis was conducted using pClamp (Molecular Devices).

### Statistics

All quantitative data in the text and figures are presented as mean±s.e.m. unless otherwise stated. Significance was calculated using multivariate ANOVA and unpaired or paired Student's *t*-test as appropriate.
